# A study of space creation for healing landscape design in the post-epidemic era

**DOI:** 10.3389/fpsyg.2025.1618451

**Published:** 2025-09-26

**Authors:** Qiugang Ren, Yueyue Weng, Zhixiong Hu

**Affiliations:** College of Design, Fujian University of Technology, Fuzhou, Fujian, China

**Keywords:** healing landscapes, post-epidemic era, space creation, multisensory design, environmental psychology

## Abstract

This study explores the spatial creation strategy of healing landscape design in the post epidemic era and identifies four types of key design elements: ecological environment elements, spatial organization elements, sensory experience elements, and social interaction elements through literature research and multi-case analysis. The study compares and analyzes domestic and international cases such as Jurong Lake Garden in Singapore, Cleveland Clinic Healing Garden, Shenzhen Bay Park "Garden of the Heart" and Liangzhu Hospital Healing Garden in Hangzhou and proposes strategies for healing landscape design at the city scale, community scale and site scale. It is found that successful healing landscapes need to integrate the principles of biophilic design, adaptive resilience, sensory calibration, social granularity, and cultural resonance, and provide solutions to promote public health and community resilience through multi-scale healing networks, micro-healing spaces, healing waterscapes, and resilient healing infrastructures.

## 1 Introduction

The COVID-19 pandemic has fundamentally reshaped human interactions with built environments, presenting unprecedented public health challenges while underscoring the therapeutic potential of intentional landscape design (Li et al., 2023). As societies adapt to post-pandemic realities, a growing scholarly and professional consensus acknowledges the critical role of purposefully designed outdoor spaces in promoting physical health, psychological resilience, and social cohesion (Panagopoulos et al., 2020). This paradigm shift has positioned healing landscapesas a central focus in contemporary landscape architecture, driving the development of evidence-based design frameworks to address the collective trauma caused by global health crises.

Therapeutic landscape design integrates spatial configuration, ecological systems, multisensory elements, and social infrastructure to create environments that actively facilitate human restoration. Moving beyond conventional aesthetic paradigms, these landscapes demonstrate measurable benefits, including the reduction of physiological stress markers, enhancement of immune system function, and reinforcement of community resilience (Panagopoulos et al., 2020; Lin et al., 2022). Such attributes have become increasingly critical in a post-pandemic world characterized by heightened isolation and uncertainty.

The conceptual framework of healing landscapes is grounded in interdisciplinary research spanning environmental psychology, public health, neuroscience, and cross-cultural healing traditions. Substantial empirical evidence confirms that exposure to well-designed natural environments yields measurable physiological benefits, including reduced cortisol levels, normalized blood pressure, enhanced cognitive restoration, and improved emotional regulation. Beyond individual well-being, these therapeutic effects contribute to broader public health objectives—ranging from disease prevention and health promotion to healthcare cost reduction—factors that now hold significant weight in post-pandemic urban policy and planning discourse.

This study examines the spatial syntax of healing landscapes in the post-COVID era, analyzing key design parameters that enable therapeutic functionality across multiple scales and contexts. Through comparative analysis of international exemplars and Chinese case studies, the research develops evidence-based design principles applicable to urban, community, and site-specific interventions (Panagopoulos et al., 2020; Razmara et al., 2021). The resulting findings advance theoretical discourse in therapeutic landscape design while providing practical guidance for creating resilient spaces that address both emergent and persistent threats to societal well-being.

## 2 Healing landscapes: key design elements for post-pandemic recovery

### 2.1 Ecological environment elements

The ecological environment constitutes the foundation of healing landscape design, offering essential natural settings that enhance therapeutic outcomes in post-pandemic contexts (Menatti and Casado da Rocha, 2016). Key elements include the biophysical features of the land surface, which directly influence human physiological and psychological responses, thereby fostering environments conducive to restoration and healing. In the aftermath of the COVID-19 pandemic, integrating ecological considerations into landscape architecture has become increasingly crucial. Studies indicate that well-designed green landscapes not only mitigate the adverse mental health effects of confinement but also improve physical well-being by providing high-quality environments (Zhang et al., 2021).

Biodiversity is a critical ecological component in landscape restoration and has been shown to offer greater therapeutic benefits than environments with lower species richness (Lai et al., 2020; Ferrando et al., 2023). Vegetation diversity—particularly when consisting of native species adapted to local conditions—enhances landscape resilience and elicits multisensory experiences that foster positive emotional responses. Empirical evidence indicates that the careful selection of plant species, such as flowering plants, medicinal herbs, and fragrant varieties, significantly increases the therapeutic potential of healing landscapes by stimulating multiple sensory modalities (Browning et al., 2014). Moreover, the intentional incorporation of diverse vegetation layers—including canopy trees, understory species, shrubs, and groundcovers—creates microhabitats that support wildlife while providing varying levels of enclosure and visibility, both of which have demonstrated psychological benefits (Wang et al., 2022; Chen et al., 2023).

**Figure 1:**
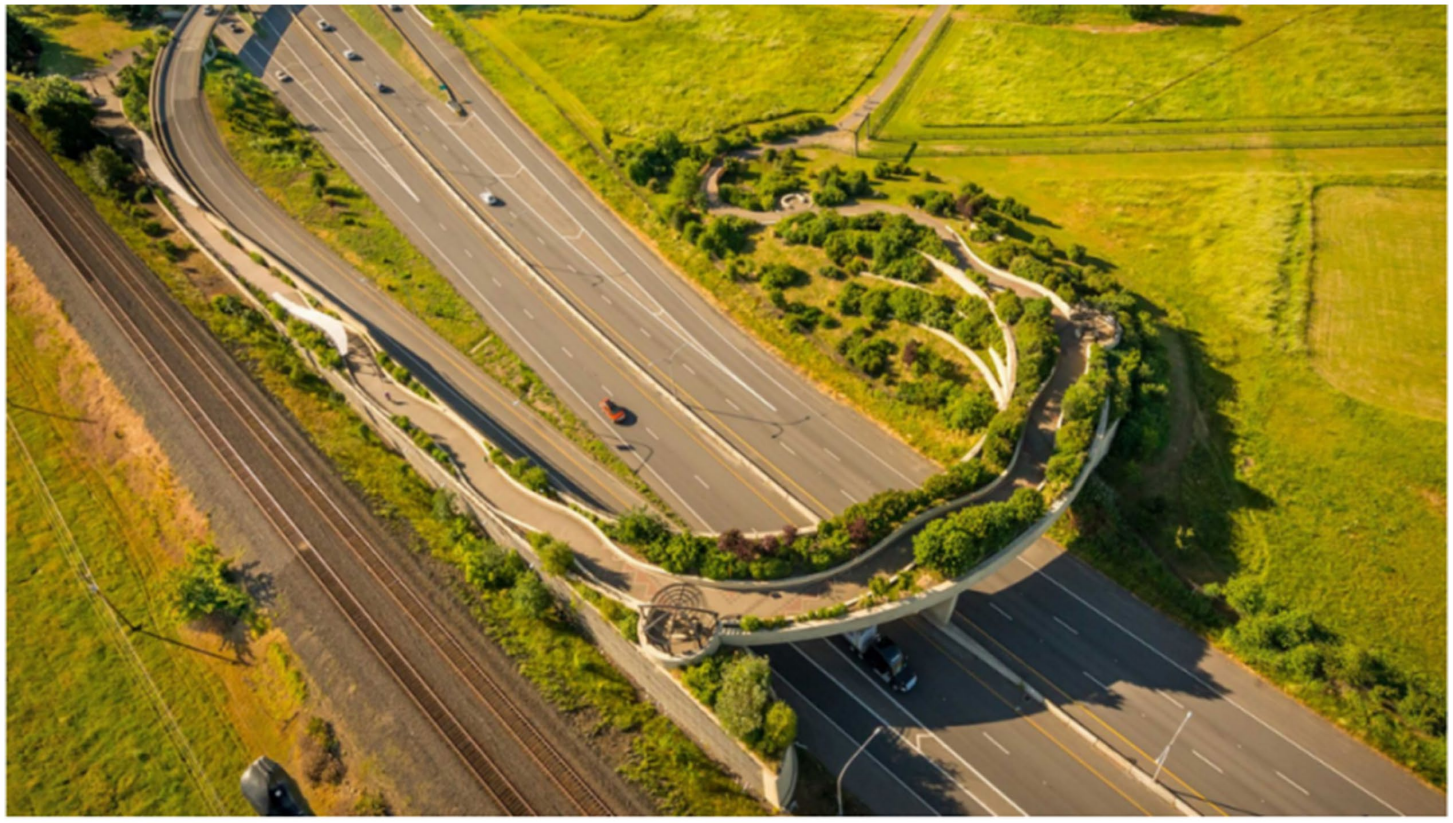
Biodiverse landscape design with layered planting structure.

As shown in Figure 1, the biodiverse landscape design with a layered planting structure exemplifies an ecological approach to healing landscapes. The aerial photograph depicts a road interchange transformed into a vegetation-rich buffer zone through strategic planting. This design demonstrates how ecological healing landscapes can be integrated into highway and railway infrastructure. By establishing a hierarchical arrangement of plant layers—from groundcovers and shrubs to tall trees—designers created a complete plant community within a limited space.

This layered planting approach not only offers visual appeal but also generates diverse microhabitats that attract birds, butterflies, and beneficial insects, thereby strengthening biodiversity. The curved pathways and vegetated boundaries illustrate how spatial definition can be achieved through topographical variation, creating spaces that are simultaneously open and private, in line with the prospect–refuge theory discussed in the text. This case demonstrates how monotonous transportation infrastructure can be transformed into healing landscapes that integrate ecological functions with psychological restorative value.

The design exemplifies the importance of ecological environment elements in healing landscapes as emphasized in the text, demonstrating how landscape design can simultaneously serve ecological functions, aesthetic values, and human health dimensions.

Water features constitute a vital ecological element in healing landscape architecture, supported by extensive research highlighting their disease-preventive and health-promoting benefits. Incorporating elements such as reflective pools, meandering streams, and interactive fountains fosters multisensory engagement through visual, auditory, and tactile stimuli. Studies indicate that water features are particularly effective in enhancing mental well-being within therapeutic gardens (Browning et al., 2014). In post-pandemic landscape design, their significance has been further emphasized, as they are associated with notions of purification, renewal, and psychological cooling, all of which play a crucial role in reducing stress and alleviating anxiety (Zhang et al., 2021).

Topographic variety constitutes a third essential ecological feature of therapeutic landscapes, offering diverse opportunities for spatial experiences and body–environment interaction. Purposefully designed landforms can create sheltered microclimates, shape visual horizons, delineate spaces for distinct activities, and provide physical challenges that support both mental and physical restoration. Research suggests that landscapes with moderate topographic complexity are generally preferred and elicit stronger restorative responses compared with those exhibiting either very low or excessively high complexity (González-Marín and Garrido-Cumbrera, 2020; Browning et al., 2014).

Climate-sensitive design represents a core environmental consideration in the development of healing landscapes, aiming to ensure user comfort and accessibility while minimizing ecological impacts. Xeriscaping—employing native, drought-resistant vegetation adapted to local climatic conditions—exemplifies ecologically informed design for creating sustainable and restorative spaces. In the post-pandemic era, climate resilience in healing landscapes has gained heightened importance, with an emphasis on facilitating prolonged and comfortable outdoor use across varying weather conditions and seasons. This can be achieved through strategies such as fostering diverse microclimates, providing shade, and harnessing natural ventilation to maintain thermal comfort throughout the year.

The incorporation of wildlife habitats introduces an additional ecological dimension to restorative landscape architecture, with research emphasizing the psychological benefits of human interaction with non-human species. Landscapes designed to attract birds, butterflies, and beneficial insects foster engagement and provide restorative distraction, thereby supporting attentional recovery and reinforcing a sense of connection to broader ecological systems. In the context of post-pandemic design, increasing emphasis has been placed on cultivating sensitivity to natural processes—particularly the seasonal and temporal dynamics characteristic of healthy ecosystems (Zeisel and Tyson, 2017).

The integration of these environmental elements forms the foundation upon which therapeutic landscapes simultaneously foster aesthetic value, human health, and overall well-being. In the post-pandemic era, principles of ecological design have gained renewed importance, as they address multiple dimensions of well-being—facilitating mental restoration, encouraging physical activity, strengthening social connections, and enriching ecosystems through the deliberate structuring of natural systems. Working in conjunction with spatial configuration, sensory engagement, and social interaction, ecological elements contribute to the creation of holistic healing environments capable of responding to the complex challenges of contemporary society.

### 2.2 Spatial organization elements

Spatial configuration is a critical aspect of therapeutic landscapes, providing the structural framework necessary to realize restorative benefits, particularly in the post-pandemic context (Souter-Brown, 2015). This concept is manifested through the three-dimensional arrangement and interaction of different spaces, creating healing experiences via the deliberate design of edges, pathways, focal points, and activity zones. Empirical research suggests that thoughtfully designed spatial configurations establish a clear hierarchy of spaces to accommodate diverse user needs, while integrating these spaces into a coherent spatial language that enhances orientation, safety, and psychological well-being.

The concept of spatial zonation serves as a fundamental organizing principle in the design of therapeutic landscapes, as research has shown that clearly defined functional areas enhance the healing potential of external environments. In the aftermath of the pandemic, the explicit separation of public, semi-public, and private zones has become increasingly important, reflecting the human need for varying levels of social interaction and personal space. Effective therapeutic landscapes require careful consideration of spatial arrangements in relation to functional requirements and their adjacency to hospital departments. Optimal therapeutic experiences are achieved when transitions between zones are both clearly delineated and fluidly integrated. Moreover, the establishment of graduated zones of privacy creates a spatial hierarchy that accommodates diverse psychological conditions and stages of recovery, enabling individuals to adjust their social engagement according to personal comfort levels.

**Figure 2:**
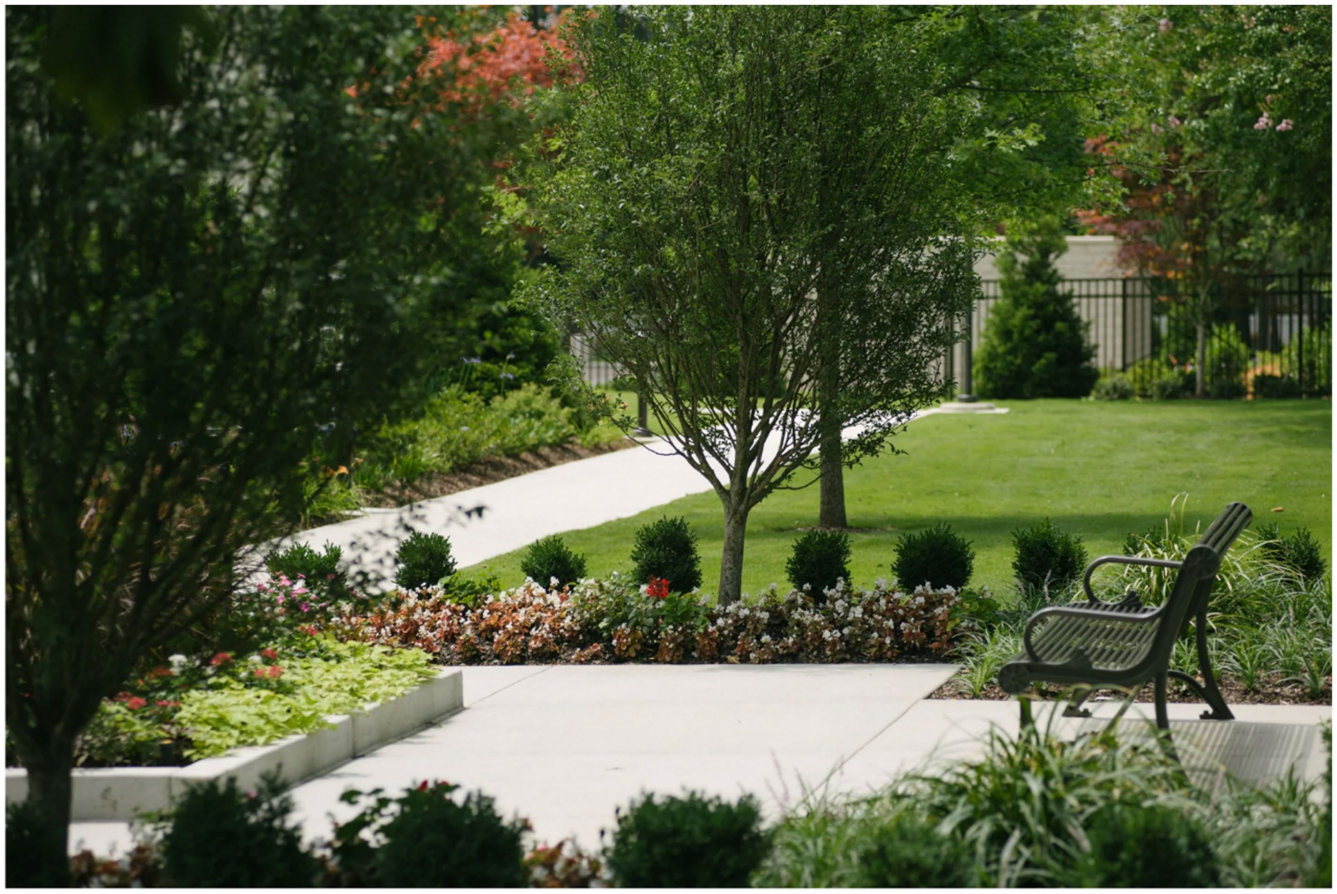
Space partitioning diagram.

As shown in Figure 2, the space partitioning diagram demonstrates the practical application of spatial organization principles in therapeutic landscapes. The illustration depicts a carefully designed outdoor environment with distinct functional areas that remain visually coherent. In the foreground, a paved seating area with a strategically placed bench provides both prospect—an open view of the lawn—and refuge—shelter offered by protective plantings. Layers of vegetation, including trees, shrubs, and flowering plants, establish natural boundaries that delineate zones without creating rigid separations.

The path system illustrates an effective circulation design, with the main walkway ensuring clear wayfinding and secondary paths branching into more intimate spaces. Varied elevations, achieved through raised planting beds and gentle slopes, further strengthen spatial definition while creating microenvironments that address diverse psychological needs. The strategic arrangement of plantings of varying sizes establishes a balance between enclosure and openness, exemplifying the prospect–refuge theory discussed in the text.

This design exemplifies how graduated privacy zones can accommodate various levels of desired social interaction, from the more public lawn area in the background to the semi-private seating space in the foreground. The permeable green barriers allow visual connection between zones while still maintaining distinct spatial identities, a design strategy that has become increasingly important in post-pandemic landscape architecture.

Circulation systems constitute a fundamental spatial component in healing environments, where networks of paths function not only as connectors but also as therapeutic elements. Empirical studies have shown that well-structured circulation patterns reduce stress by facilitating predictable wayfinding and enhancing overall mobility, thereby supporting the recovery process. Such systems may include roads, parkways, drives, trails, walkways, parking areas, and canals, with their characteristics shaped by alignment, width, surface treatment, gradient, materiality, and supporting infrastructure. In post-pandemic design, circulation systems have been adapted with wider pathways to accommodate social distancing while preserving connectivity between complementary programmatic spaces (Souter-Brown, 2015; MacKenzie and Rakel, 2022). The incorporation of primary, secondary, and tertiary pathway hierarchies creates a coherent system that ensures intuitive navigation and accommodates diverse mobility needs and circulation rates (Cooper Marcus and Sachs, 2014; Appleton, 1975).

The concept of prospect–refuge balance represents a third fundamental spatial dimension influencing the restorative potential of landscapes. Rooted in evolutionary psychology, this theory posits that humans possess an innate preference for environments combining expansive views (prospect) with sheltered, protected spaces (refuge). Empirical evidence indicates that landscapes maintaining a balance between these attributes provide greater restorative benefits than those dominated by either element alone. In post-pandemic contexts, the application of prospect–refuge principles has gained prominence, reflecting the increased human need for both social interaction and solitude. Strategically locating seating at the interface of open and enclosed areas creates psychologically supportive settings that accommodate both extroverted and introverted modes of restoration (Dosen and Ostwald, 2016; Lu et al., 2025).

Edge conditions constitute another critical dimension in the design of therapeutic landscapes, serving as transitional elements that bridge spaces and programmatic zones (Cooper Marcus and Sachs, 2014). Empirical evidence suggests that well-defined edges enhance spatial clarity while enabling gradual transitions between contrasting zones and varying degrees of seclusion. Research further indicates that individuals prefer environments with both vertical and horizontal expansiveness, yet articulated into smaller, more intimate spaces, underscoring the importance of establishing hierarchical edge conditions. During and after the pandemic, edge zones have gained prominence as intermediary spaces that facilitate interaction among diverse users and activities. The use of permeable barriers—such as partially open screens, subtle grade changes, and vegetative thresholds—helps articulate spatial boundaries while avoiding rigid separations.

Nodes and focal points represent a fifth essential spatial element in the design of healing landscapes, functioning as orientational markers and hubs of activity that strengthen experiential connections to the environment (Appleton, 1975). Studies demonstrate that strategically positioned nodes enhance spatial awareness by providing salient reference points that reinforce wayfinding and establish destinations that encourage mobility. In post-pandemic contexts, distributed nodes—rather than centralized gathering spaces—better accommodate diverse patterns of use and adapt to evolving social distancing requirements. The integration of both stimulating and contemplative nodes addresses varied user needs, with evidence indicating that a balanced provision of socially oriented and reflective focal points maximizes therapeutic benefits across different user groups.

The integration of these spatial organization elements provides a comprehensive framework that supports healing processes through environmental experiences. In post-pandemic contexts, greater emphasis is placed on spatial adaptability and flexibility, as research indicates that environments capable of accommodating changing user needs while adhering to safety guidelines offer more sustainable therapeutic benefits. Through the careful manipulation of spatial sequences, boundaries, and transitions, designers can create healing landscapes that address the complex requirements of human recovery while fostering resilience by engaging both physiological and psychological mechanisms of healing (MacKenzie and Rakel, 2022; Lu et al., 2025).

### 2.3 Sensory experience elements

Sensory elements constitute an essential component of healing landscape architecture, engaging multiple perceptual systems to foster meaningful therapeutic interactions in post-pandemic spaces (Spence and Puccinelli, 2022). Informed by neurological mechanisms, these elements highlight how environmental stimuli shape both psychological and physiological responses, thereby enabling multisensory environments to promote recovery, restoration, and overall well-being. In the post-pandemic context, the role of sensory inputs has become increasingly significant, as research indicates that multisensory engagement mitigates the effects of prolonged isolation and virtual fatigue through embodied interaction with the built environment.

Visual elements represent a fundamental sensory dimension in therapeutic environments, with substantial evidence demonstrating that specific visual features can elicit significant healing responses. Contemporary research indicates that visual stimuli exert primary effects on psychological outcomes, with environments characterized by high plant diversity playing a particularly important role in promoting psychological recovery. The deliberate use of color, form, and lighting generates visual configurations that mitigate mental fatigue and foster positive emotional states (Bell et al., 2022). In post-pandemic contexts, visual variety and complexity have gained prominence in counteracting the monotony associated with prolonged confinement. Studies further suggest that graduated visual complexity enhances attention restoration while minimizing the risk of overstimulation (Spence and Puccinelli, 2022; Wood et al., 2022).

**Figure 3:**
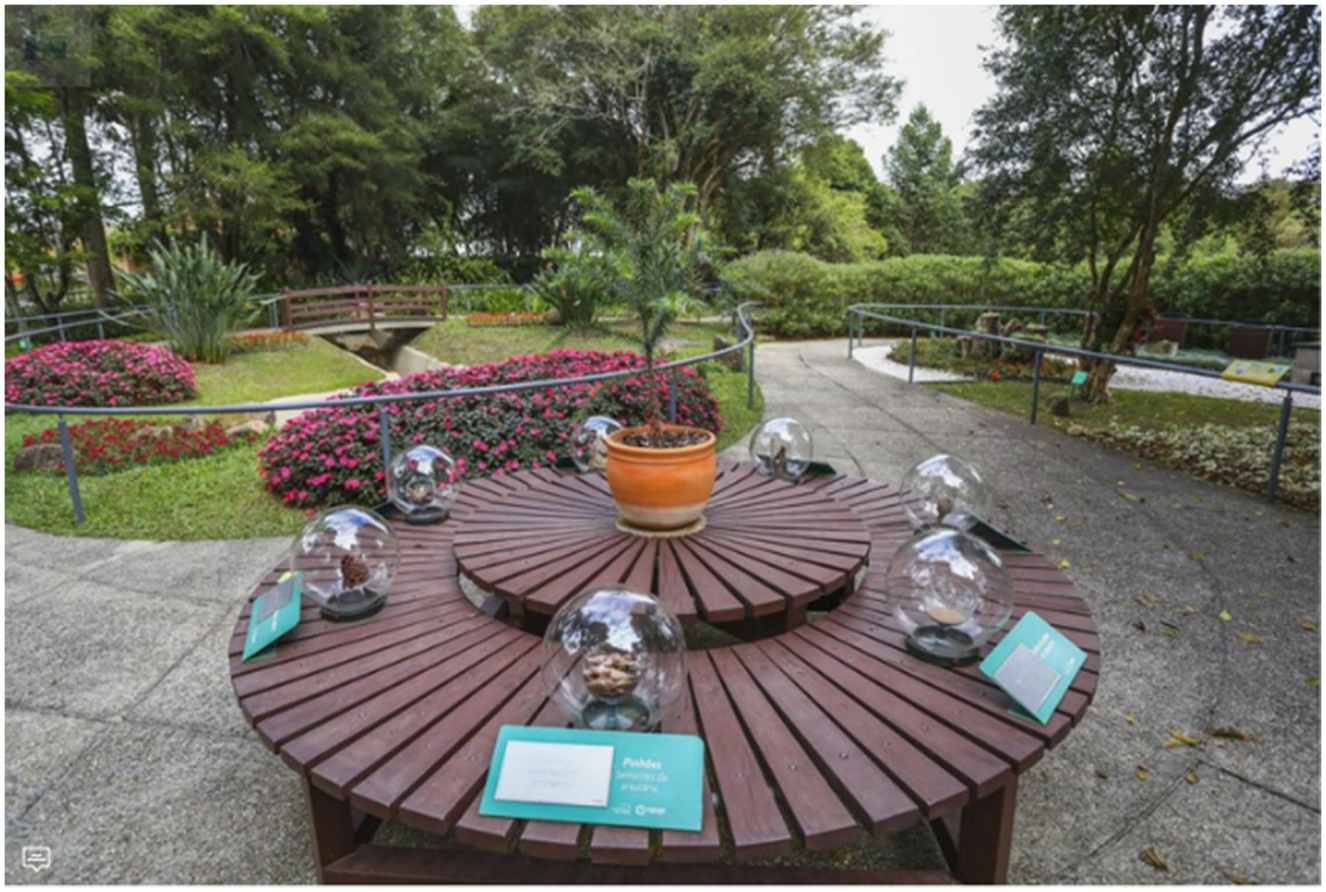
Visual elements.

As shown in Figure 3, this installation illustrates the multisensory approach within healing landscapes. The image depicts an interactive sensory exhibit designed to engage visitors across multiple perceptual dimensions. At its center, a circular wooden table displays glass domes containing diverse sensory elements, framed by vibrant flowering plants and pathways that guide movement through the space. The carefully orchestrated arrangement demonstrates how visual stimuli can be strategically integrated into therapeutic environments to foster immersive and restorative experiences.

The installation exemplifies the principles outlined in the text regarding the role of visual variety and complexity in post-pandemic healing landscapes. The colorful flowering plants establish a "high plant variety" environment shown by research to promote psychological recovery, while the circular configuration of the display creates a focal point that attracts attention and supports mental restoration. The transparent domes further stimulate curiosity and interaction, encouraging visitors to engage more deeply with the surrounding natural elements.

The setting also illustrates the integration of multiple sensory elements that extend beyond visual appeal. Auditory stimuli may be introduced through rustling leaves or water features, tactile engagement through diverse materials and textures, and olfactory stimulation through the presence of flowering plants. This multisensory approach exemplifies how therapeutic landscapes can counteract the sensory deprivation associated with periods of isolation, fostering environments that engage the full spectrum of human perception and promote holistic recovery.

Auditory elements constitute a core sensory dimension in healing landscapes, with soundscape design playing a critical role in shaping both physiological and psychological responses. Empirical research has demonstrated that natural auditory stimuli provide direct benefits for perceived restorativeness, emotional states, and user preferences. The incorporation of biophonic sounds—such as birdsong, flowing water, and rustling leaves—creates acoustic environments that alleviate stress by regulating indicators such as heart rate, blood pressure, and cortisol levels. In post-pandemic contexts, the design of healing landscapes increasingly emphasizes the integration of acoustic barriers with sound-enhancing features to mitigate urban noise pollution and amplify restorative natural soundscapes, thereby creating acoustic refuges that support recovery from sensory overstimulation.

Tactile elements represent a tertiary sensory dimension essential to therapeutic engagement with landscapes, activating the haptic system through diverse surface textures, thermal contrasts, and tactile sensations. Research indicates that tactile stimulation activates the parasympathetic nervous system, fostering relaxation and recovery from stress. While architecture has traditionally been dominated by visual considerations, designers are increasingly incorporating other senses—particularly sound and touch, including proprioception, kinesthesis, and vestibular perception—into their work. The integration of varied textural experiences through plant selection, materiality, and topographical variation creates environments that encourage bodily engagement and embodiment. In the post-pandemic context, tactile elements have gained renewed significance as a response to touch deprivation during prolonged isolation, with studies showing that diverse tactile stimuli enhance emotional well-being.

Olfactory elements represent a distinctive sensory dimension in healing landscapes, with research demonstrating their strong influence on emotional responses and place-based memories (Pallasmaa, 2012). Scents can evoke memories and assist navigation (Wood et al., 2022), making them a powerful tool for shaping meaningful experiences across different landscapes. The intentional use of fragrant flowers, seasonally shifting aromas, and scent-based zones establishes olfactory conditions that elicit positive emotional responses and foster the formation of site-specific memories. In post-pandemic contexts, olfactory elements have gained renewed importance due to their capacity to influence emotions directly, independent of cognitive appraisal. Studies indicate that natural plant fragrances can effectively reduce anxiety and elevate morale, thereby enhancing the therapeutic quality of healing environments.

The integration of sensory elements enriches healing environments by engaging the full spectrum of human perceptual capacities in a synergistic manner, thereby facilitating recovery through multisensory interaction. As designers shift from creating "therapeutic landscapes" to developing therapeutic "sensescapes" they establish more inclusive environments that address the diverse needs and preferences of users. This multisensory approach is particularly salient in the post-pandemic context, where healing environments can help individuals restore sensory capacities diminished by prolonged isolation and reliance on virtual interaction.

### 2.4 Social interaction elements

Social interaction elements represent a fundamental dimension of healing landscape design, fostering interpersonal connections and community engagement that support recovery and well-being in post-pandemic contexts (Teig et al., 2021). These elements recognize the inherently social nature of healing and the critical role of social connections in enhancing therapeutic outcomes across diverse user groups (Bell et al., 2023). Empirical evidence indicates that well-designed social spaces can alleviate isolation, cultivate a sense of belonging, and strengthen support networks essential for mental health resilience.

**Figure 4:**
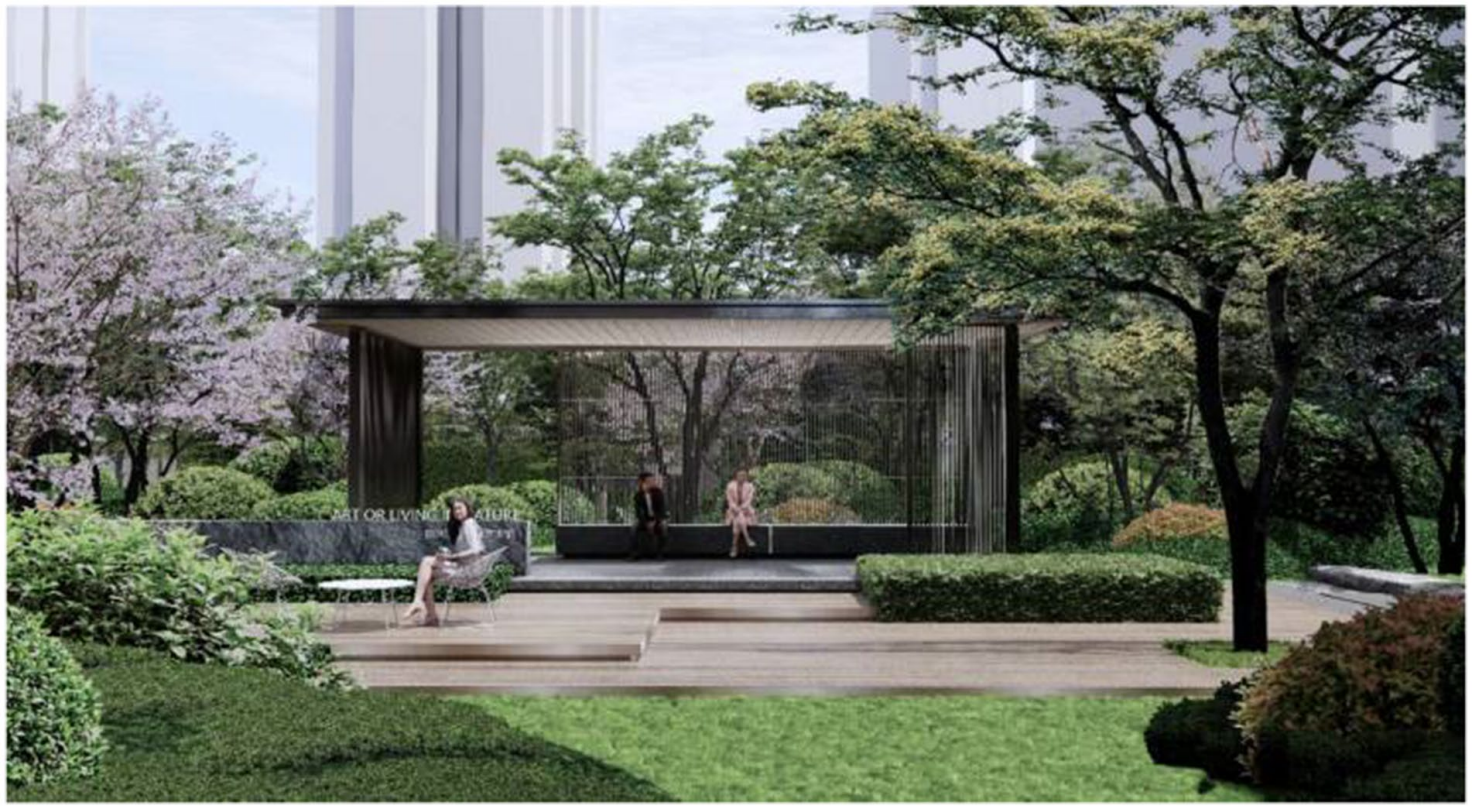
Public activity area.

As shown in Figure 4, this public activity area exemplifies the social interaction elements central to healing landscape design. The image depicts a contemporary pavilion designed to accommodate varying levels of social engagement while maintaining a strong relationship with the natural setting. The transparent structure functions as a focal point, establishing a shared gathering space that preserves visual and spatial connectivity with the surrounding landscape. The stepped platform creates gradated zones of sociability, enabling more private interactions along the edges and fostering communal activities within the central pavilion.

This design illustrates the principles of flexible social spaces discussed in the text, accommodating varying levels of social interaction. The pavilion’s transparent walls establish permeable boundaries that preserve visual connectivity while offering subtle separation—a strategy that proved particularly valuable during the pandemic, when maintaining social distance was necessary yet human connection remained essential. Surrounding trees and vegetation contribute both aesthetic value and a sense of enclosure, thereby enhancing psychological comfort.

This space demonstrates how thoughtful landscape architecture can strengthen the social dimensions of healing by fostering meaningful human interaction while accommodating individual needs for varying degrees of privacy. Its adaptable design enables use as both a performance venue and an informal meeting area, exemplifying how multipurpose spaces in healing landscapes can promote community engagement while remaining responsive to evolving public health requirements.

Gradated zones of sociability form an essential component of therapeutic landscapes, enabling environments to accommodate varying levels of social interaction and seclusion in an orderly manner. Empirical studies suggest that opportunities for social interaction and peer support are critical mechanisms through which therapeutic environments enhance mental well-being. The intentional configuration of seating clusters, shared areas, and secluded spaces provides flexibility in social engagement, allowing adaptation to users’ psychological states and rehabilitation needs. In post-pandemic contexts, the role of gradated zones has become increasingly significant, facilitating social distancing while simultaneously supporting human connection (Dushkova and Ignatieva, 2020).

The sharing of spaces constitutes a fundamental aspect of social growth, offering opportunities for collective participation that enhance both mental and physical well-being. Evidence indicates that neighborhood gardeners are more likely to develop social networks with neighbors, engage in civic activities, and thereby strengthen community ties. The integration of shared garden spaces, outdoor educational settings, and adaptable meeting areas creates environments that foster purposeful social interaction alongside skill development and knowledge exchange. In response to the pandemic, therapeutic landscapes increasingly incorporate multipurpose spaces that can be adapted to evolving public health measures while continuing to facilitate participatory communal engagement.

The principle of cultural inclusiveness represents a tertiary social element in healing landscapes, emphasizing that successful therapeutic spaces must reflect the diverse cultural backgrounds and needs of their users. Empirical evidence demonstrates that landscapes incorporating cultural references and customs significantly enhance participation and therapeutic outcomes among diverse groups. Research further highlights that outdoor spaces can foster rehabilitative healing, inclusive communities, and self-empowerment when designed with sensitivity to cultural contexts. The integration of culturally relevant plantings, design motifs, and spatial arrangements thus produces landscapes that honor cultural identities, strengthen sense of place, and enrich user interaction with therapeutic environments.

Intergenerational design elements represent a vital social dimension in healing landscapes, as they facilitate interactions across age groups and foster shared social experiences and knowledge exchange (Zhao et al., 2024). Evidence shows that spaces intentionally designed to encourage intergenerational engagement not only strengthen social bonds but also address the diverse needs of different user populations (Rammo et al., 2022). Studies further indicate that vegetable gardens, for example, provide meaning, connection, enjoyment, and tranquility for individuals across age ranges. The integration of universally accessible features, activity spaces appealing to multiple age groups, and areas that invite communal storytelling creates landscapes that bridge generational divides while promoting mutual support and understanding (Croeser et al., 2024).

## 3 Applications of healing landscapes in spaces

### 3.1 Applications in urban public spaces

The concept of therapeutic design within therapeutic landscapes is increasingly integrated into urban public spaces, as cities recognize its potential to address complex health challenges in post-pandemic contexts. Such interventions transform neglected areas into multifunctional environments that foster physical well-being, support mental restoration, and encourage social interaction. Case studies demonstrate that intentional design can create urban sanctuaries that mitigate stress and sensory overload commonly associated with city life.

The High Line in New York exemplifies the successful integration of healing landscapes into urban infrastructure. This 1.5-mile linear park transforms an abandoned railway into a therapeutic corridor composed of diverse sensory spaces and restorative zones. The intentional design of seasonal plant variations fosters a dynamic and stimulating environment that engages users on multiple sensory levels. Furthermore, the spatial diversity—from intimate social niches to expansive observation platforms—enables individuals to shape their social interactions and sensory experiences according to personal preferences. This adaptability illustrates the principle of graduated sociability, a core tenet of healing landscape design.

**Figure 5:**
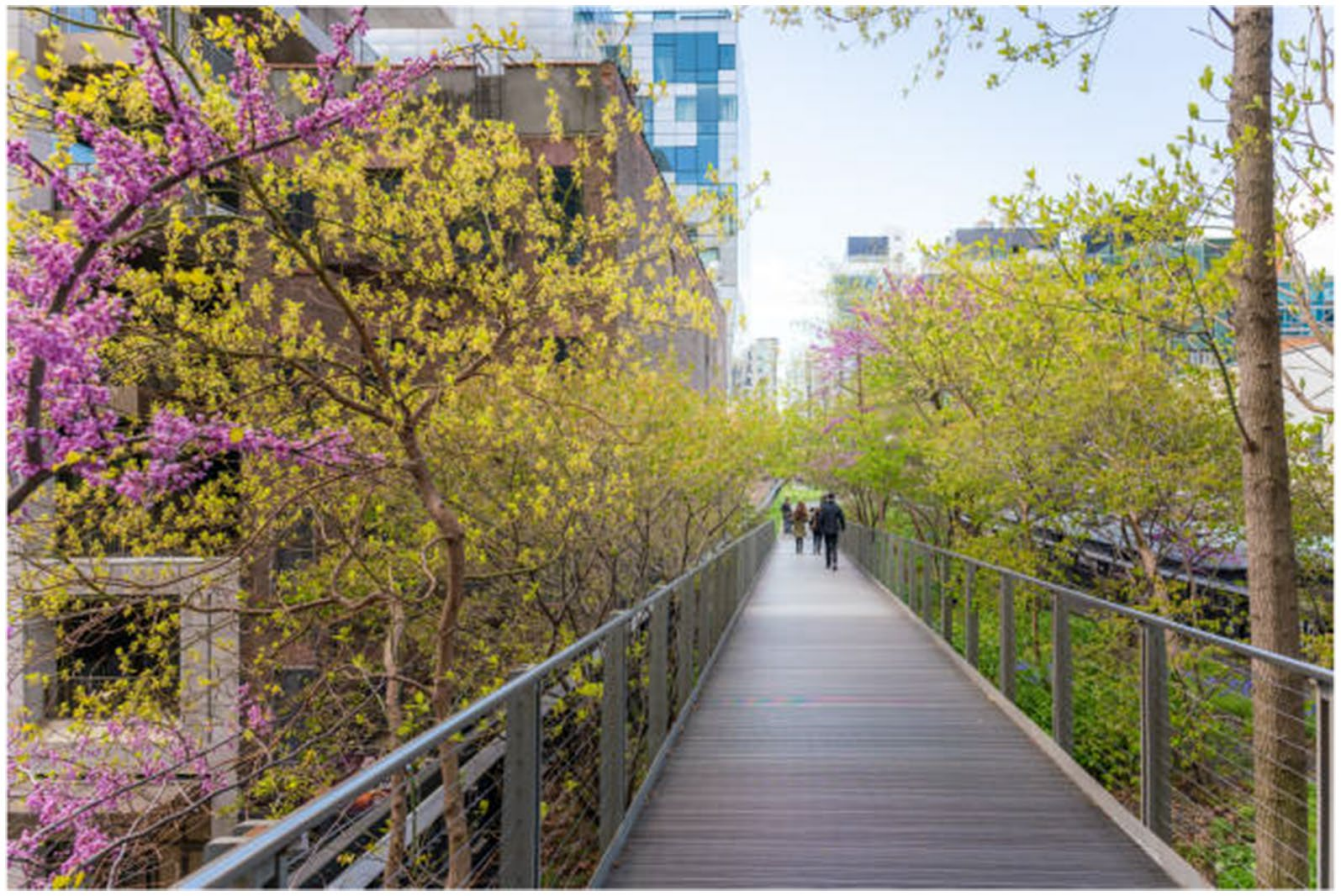
High line park.

As shown in Figure 5, the High Line Park exemplifies urban healing landscape integration in post-pandemic city design. The image captures an elevated wooden pathway flanked by vibrant flowering trees in spring bloom, creating a striking visual corridor through the urban environment. Pink and yellow blossoms frame the walkway, offering a sensory-rich experience that contrasts with the surrounding city buildings visible in the background. This elevated linear park demonstrates how abandoned infrastructure can be transformed into therapeutic green spaces that serve multiple healing functions.

The design exemplifies the concept of a healing corridor discussed in the text, with the elevated boardwalk offering a clearly defined circulation route that guides visitors through diverse sensory experiences. The seasonal flowering trees highlight the intentional incorporation of temporal change in planting design, generating dynamic visual interest that evolves throughout the year. This strategy creates the "stimulating and dynamic environment" referenced in the text, engaging users across multiple sensory dimensions.

The linear configuration establishes a distinctive journey experience, enabling visitors to transition between spatial zones while simultaneously maintaining a sense of prospect—views extending toward the city—and refuge—protection offered by the tree canopy. This calibrated balance of openness and enclosure exemplifies how urban healing landscapes can alleviate urban stressors while preserving a tangible connection to the surrounding city fabric. In doing so, it demonstrates the effective application of healing landscape principles within high-density urban environments.

Urban pocket parks represent effective small-scale applications of healing landscape principles, demonstrating that therapeutic design can yield significant benefits even within limited spatial contexts. These compact green spaces facilitate accessible engagement with nature in densely urbanized environments while simultaneously supporting biodiversity and offering settings for contemplation and social interaction. When strategically located near hospitals, clinics, business districts, and residential areas, pocket parks integrate seamlessly into daily routines, thereby maximizing their therapeutic impact across diverse populations.

The design principles of healing plazas reimagine traditional urban plazas by transforming them from predominantly hardscaped environments into biophilic settings that foster multisensory engagement. Contemporary plaza designs increasingly incorporate elements such as water features, diverse vegetation, flexible seating arrangements, and natural materials to enhance physical well-being and encourage social interaction. These redeveloped plazas illustrate how healing landscapes can preserve the social and cultural functions of urban gathering spaces while simultaneously expanding their capacity to support both mental and physical health.

Waterfront revitalization projects present particularly significant opportunities to apply healscape principles in urban contexts. Such initiatives harness the inherent therapeutic qualities of waterfront environments while simultaneously rehabilitating post-industrial sites to enhance community well-being. By integrating ecological restoration with therapeutic space, these projects foster environments that support both ecological health and human wellness. Linear configurations create extensive corridors that encourage mobility and physical activity, whereas varied spatial arrangements provide settings for social interaction as well as quieter areas for contemplation, thereby accommodating diverse therapeutic needs.

### 3.2 Applications in community scale landscapes

The integration of therapeutic landscapes into community spaces serves as a crucial intermediary, bridging public urban environments with personal domains and providing consistent access to supportive services aimed at promoting well-being. In the post-pandemic era, public spaces have assumed an increasingly therapeutic role by mitigating the effects of collective trauma and social isolation through the creation of safe, open, and restorative environments. These communal spaces integrate ecological, spatial, sensory, and social dimensions into cohesive systems of healing landscapes that foster resilience and holistic well-being.

**Figure 6:**
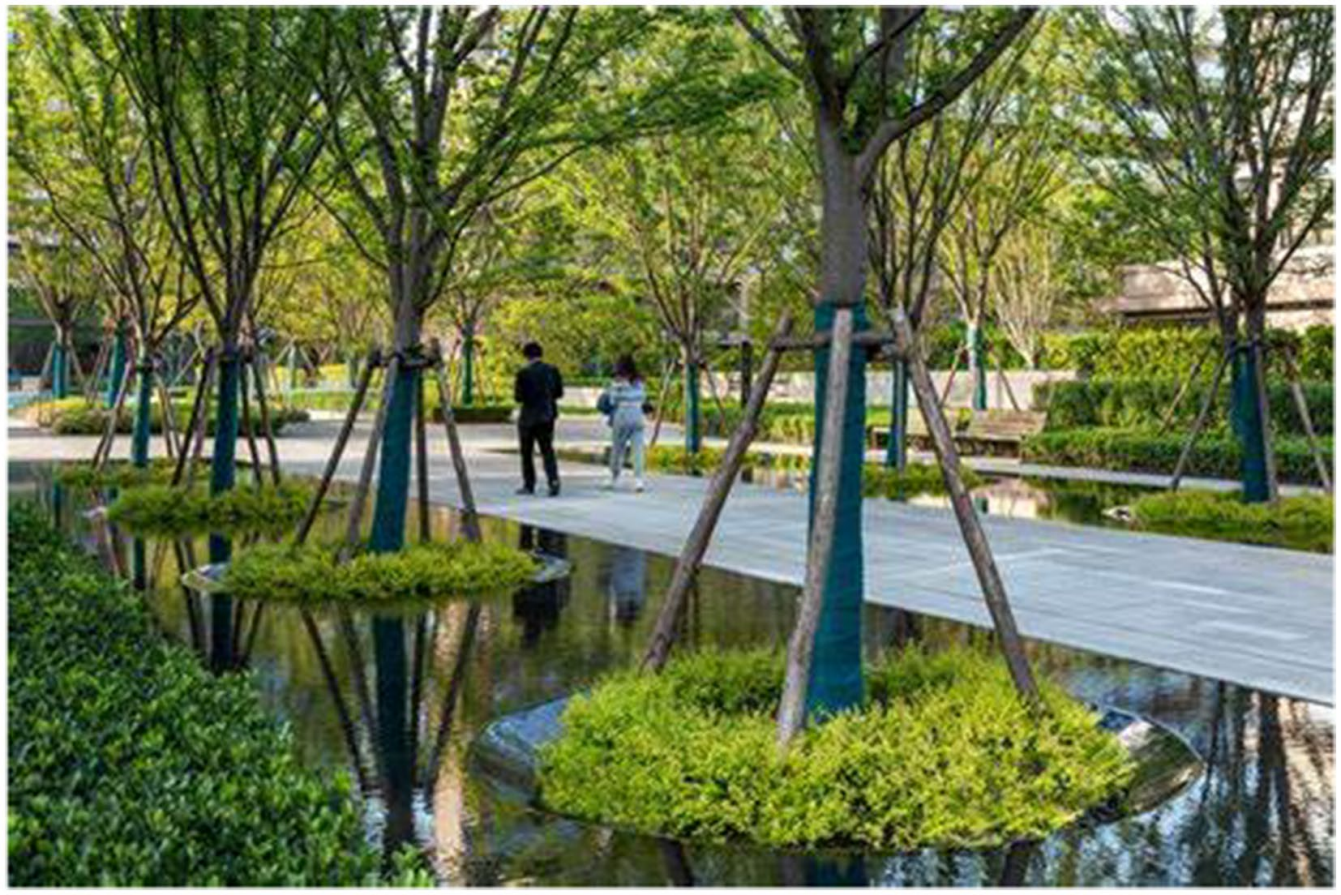
Healing integration of community waters and stack systems.

Water elements occupy a central role in therapeutic landscapes across many communities, as illustrated in Figure 6, where the interplay between reflective water bodies and vertical vegetation amplifies spatial experience. Beyond creating visual expansion through reflective qualities, water features stimulate multisensory engagement by incorporating auditory effects and contributing to climatic moderation. Empirical research has demonstrated that aquatic landscapes possess a strong capacity to reduce stress and enhance overall well-being among community residents. A well-designed water feature achieves a balance between ecological performance and aesthetic appeal, offering a safe, water-centered experience supported by structural frameworks while maintaining equilibrium between openness and seclusion.

Hiking networks within community spaces function as linear healing corridors that connect residential areas with diverse community resources. As illustrated in the diagram, trestle structures not only facilitate physical engagement but also influence movement tempo and the potential for social interaction by shaping pathways that guide visual attention and bodily motion. An effective healing trail system incorporates water features and vegetation to establish clear boundary conditions and thresholds, thereby enriching users’ experiences of nature and enhancing the therapeutic value of the trails.

Vertical greening elements play an important role in spatially limited environments, as they generate a natural dome effect by the strategic placement of tall trees that provide shade and a sense of visual closure. This design element enhances the gravity and formality that pervade the space, while at the same time, it induces a sense of connection to the outside and provides for the universal human desire for a "forest bathing" atmosphere. The use of raised pathways and water elements enables these spaces to define a varied hierarchy within the space, allowing for emotional engagement in small dimensions to provide for a range of individual reflection and social interaction needs.

### 3.3 Applications in healthcare and rehabilitation environments

Health and rehabilitation centers represent focal sites for the application of healing landscapes, where landscapes are conceived as integral components of comprehensive treatment strategies rather than as cosmetic additions to hospital settings. In the post-pandemic context, hospitals and healthcare facilities increasingly acknowledge the critical role of external spaces in supporting patient recovery, enhancing the well-being of healthcare professionals, and improving overall care quality, thereby driving the adoption of advanced healing landscape design approaches.

**Figure 7:**
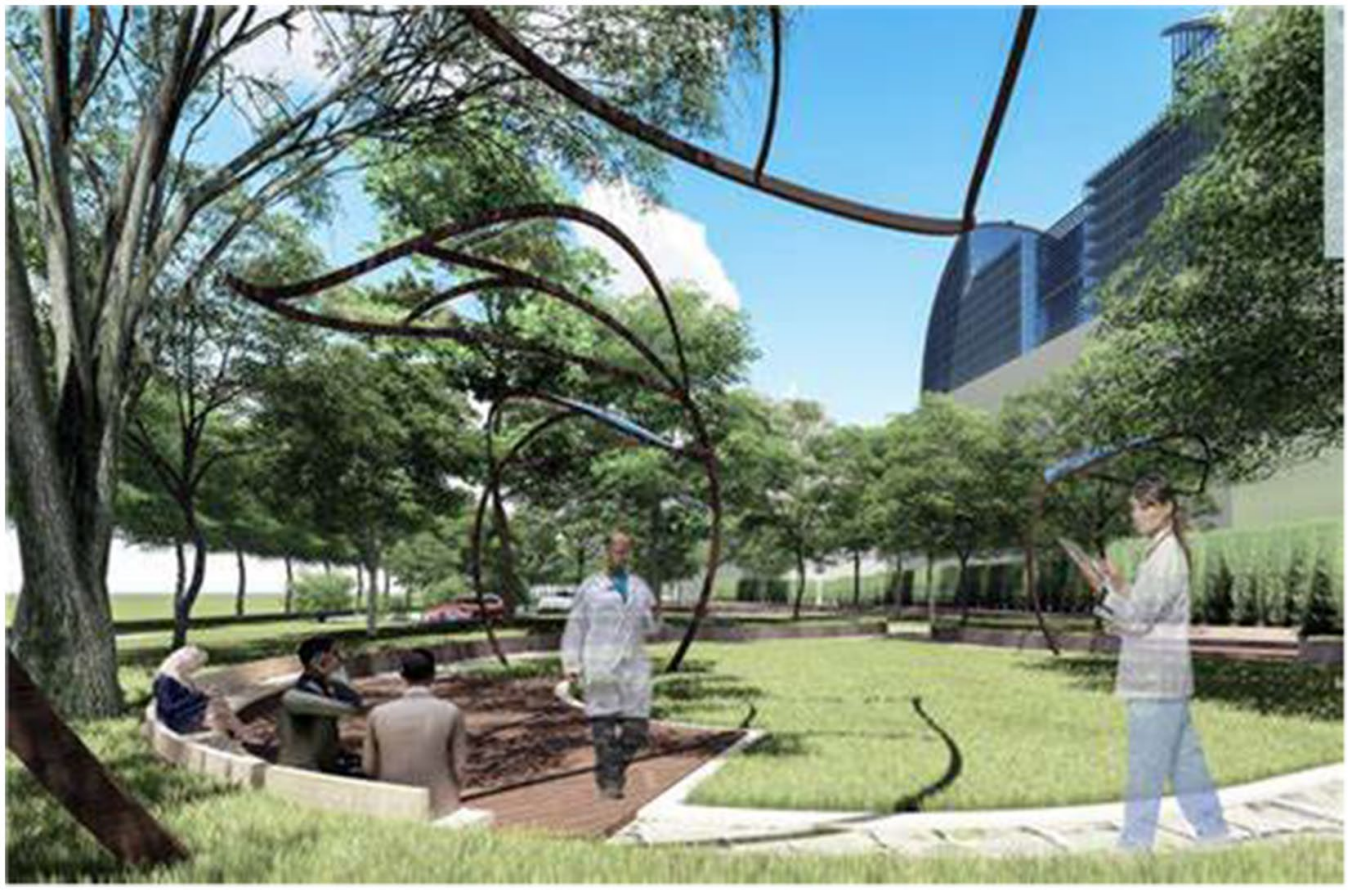
Semi-open healing rest area design in healthcare institutions.

As illustrated in Figure 7, the configuration of therapeutic landscapes in contemporary healthcare environments emphasizes the creation of universal spaces that encourage social interaction while maintaining strong connections with nature. The integration of semi-open resting areas within architectural design fosters opportunities for social engagement while preserving privacy and ensuring security—conditions particularly vital in post-pandemic healthcare contexts. The inclusion of tree canopies provides natural shading that mitigates direct sunlight and contributes to favorable microclimatic conditions. Meanwhile, the undulating forms of tree branches enrich visual perception, enhancing both the dynamic and rhythmic qualities of the healing space.

The conceptual design of therapeutic landscapes within healthcare settings follows the principle of "progressive stress reduction," thus creating routes that divert patients from busy clinical areas to calm environments that promote contemplation. The space design gives users the ability to select best environments that best suit their mental conditions and includes communal spaces (like the shared relaxation space demonstrated in the adjacent photo) and private secluded spaces. Empirical research has found that a high level of voluntary choice significantly increases the sense of patients' control and autonomy and thus contributes to the healing process.

The complexity of habitats determines to a great extent the architectural aesthetic in hospitals, ensuring intense sensory interaction and biodiversity via varied strata of vegetation and terrain. The vegetation patterns with varying elevations and textures not only ensure visual beauty but also form varied ecosystems with the ability to attract bird populations and beneficial invertebrates and hence provide opportunities for natural interventions and positive distractions to patients.

The therapeutic landscapes in hospitals strongly reflect the need to conform to universal design standards to enable persons with different capacities to have equal access to natural environments. The research provides evidence to support the notion that well-planned paths and adaptive seating spaces enable patients with mobility impairment to have equal participation in outside spaces while experiencing the healing benefits from exposure to the outdoors. At the same time, clear wayfinding and signage systems minimize cognitive loads to ensure a secure and trustworthy interaction between the vulnerable person and the space.

### 3.4 Applications in workplaces and educational spaces

**Figure 8:**
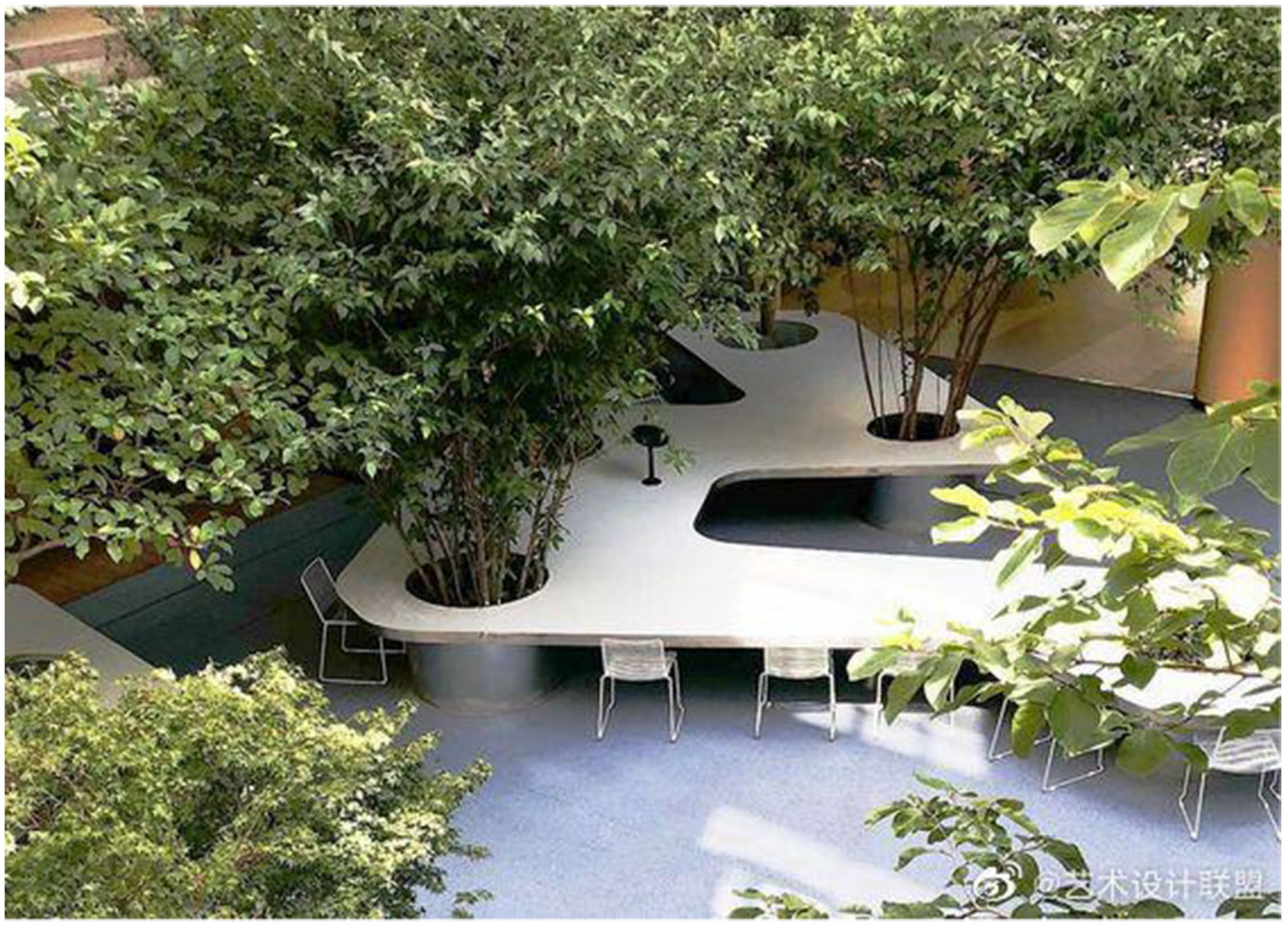
Biophilic transitional zones in workplace environments.

**Figure 9:**
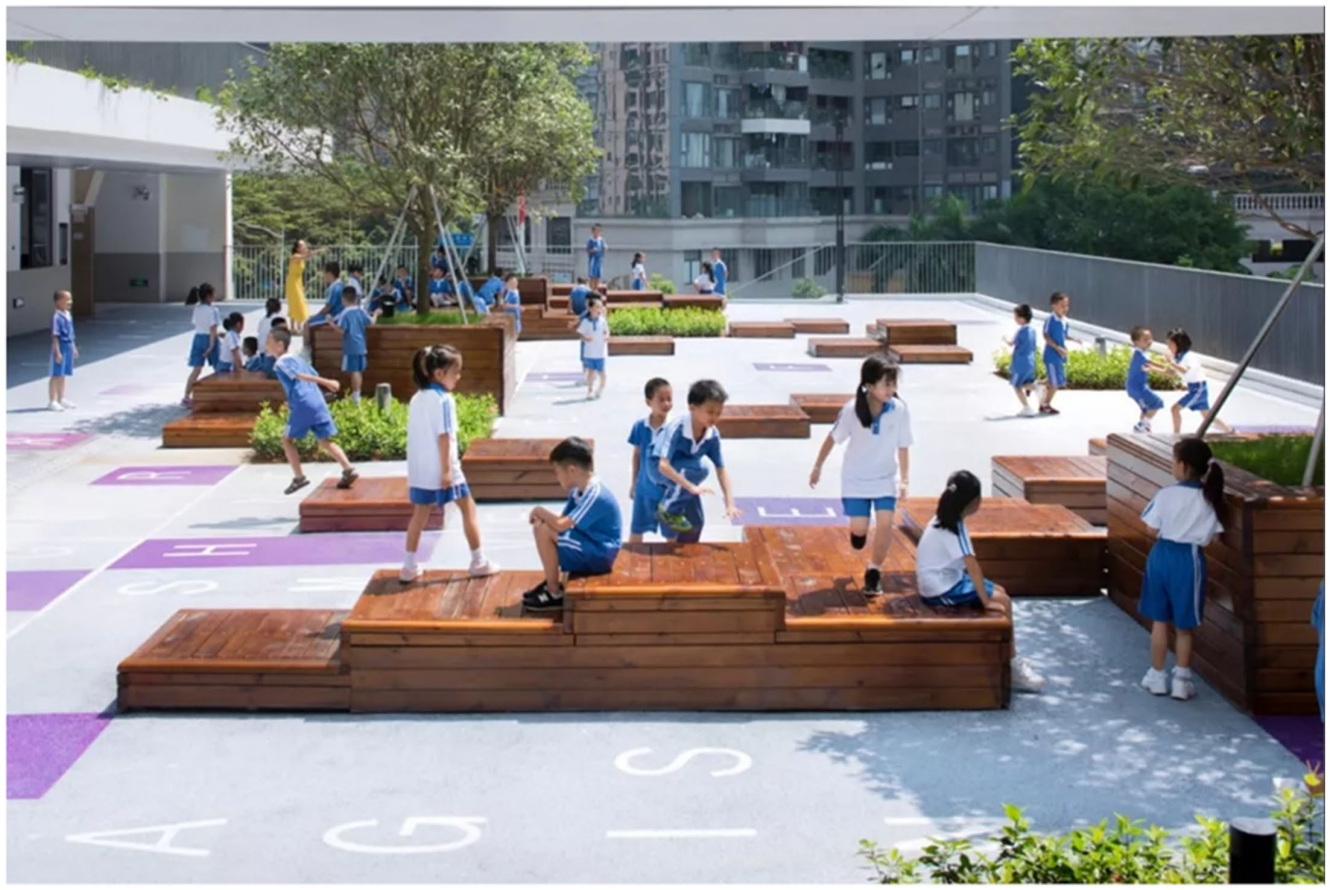
Modular educational healing landscape with flexible learning platforms.

The integration of therapeutic landscapes in educational and working environments is a significant development in the design and conceptualization process of institutional landscapes after the pandemic. As seen in Figures 8, 9, contemporary thought regarding such functional landscapes accords equal value to mental well-being and practicality in designing integrated environments that improve cognitive functions with equal attention to social development and improved overall bodily well-being.

Schools have considerably embraced healing landscapes with recognition of their ability to enhance educational performance and promote the holistic development of students. Figure 9 describes a new model for educational outdoor spaces in which traditional rigid play spaces have been redesigned to be flexible and multifunctional learning landscapes. The modular wood platforms provide topographical variety to promote varied body interaction—sitting, climbing, and social interaction—coupled with defining spaces that can be allocated to different functions and group numbers. Incorporating vegetation intentionally in the breaks creates natural shade and visual relief and creates microclimates to improve microclimates' comfort levels with educational activities outdoors.

The elements of space disposition found here educational facility demonstrate a thoughtful balance between form and flexibility through clearly defined areas that simultaneously support free and spontaneous usage patterns. The philosophical design approach recognizes educational spaces' need to support structured activities and independent exploration alike, an aspect that has come to be widely important in post-pandemic educational environments with flexible learning methods becoming a much-deemed priority. The range from communal to half-private spaces found in the varying platform configurations allows individuals to opt for the social interaction they prefer, thus addressing varied learning tastes and psychological comfort levels.

In workplaces, as shown in Figure 8, healing landscapes increasingly serve as go-between spaces linking indoor and outdoor environments, thus enabling workplace transitions to permit rejuvenating pauses from intense work while also encouraging spontaneous collaboration. The courtyard design showcases how drought-resistant vegetation and natural material use can establish microhabitats that boost sensory diversity and advance a biophilic connection amidst highly structured architectural spaces. The circulation routes provided by the design promote breaks in the workday that have been shown to be vital to both intellectual function and physical well-being.

The purposeful inclusion of restoration landscapes in educational and professional settings provides substantial benefits in multiple areas. Educationally, research has illustrated the benefits to attention levels from the inclusion in school and university settings of well-planned outdoor learning spaces, with reduced behaviors and heightened creativity, in addition to improving interpersonal relations among students. Professionally, restoration landscapes bring about improved job satisfaction levels, promote collaborative working environments, and lower absenteeism—propositions that have emerged strongly in the aftermath of the global pandemic and the resultant prioritization of employee well-being as a core organizational goal.

## 4 Case studies analysis

### 4.1 Research methodology and case selection

This study adopts a multiple-case study approach to systematically examine the spatial attributes of healing landscapes and to distill key elements and functional guidelines for their design in the post-pandemic era through comparative analysis of both foreign and local cases. Case selection followed four criteria to ensure methodological rigor and representativeness: (1) projects initiated or substantially redesigned after the COVID-19 outbreak (2019 onward) to embody post-pandemic design principles; (2) explicit emphasis on enhancing users’ physical and mental well-being as a primary design goal; (3) completion and operation for at least 1 year to allow sufficient accumulation of user experience and evaluative data; and (4) diversity in geographic regions, cultural contexts, and functional types to broaden the scope and applicability of findings. Analytical depth was enhanced through a structured framework encompassing three interrelated dimensions: physical–spatial characteristics (layout, organization, elements), user experience metrics (behavioral mapping and satisfaction surveys), and therapeutic performance indicators (where pre- and post-intervention data were available). Data collection combined systematic document review (project briefs, design specifications, post-occupancy evaluations), structured site observations (capturing usage patterns, behaviors, and environmental qualities over time), semi-structured interviews with designers, managers, and users (2–3 per case), and available quantitative performance data. Finally, a systematic cross-case comparison was conducted using standardized criteria to evaluate pandemic-responsive features, therapeutic outcomes, cultural adaptation strategies, and innovations in spatial programming, thereby identifying both context-specific insights and transferable design principles.

This research employs a mixed-methods approach that integrates quantitative assessment using the Healing Landscape Assessment Matrix (HLAM) with qualitative analysis based on grounded theory. The HLAM provides a comprehensive evaluation framework across four dimensions: (1) ecological environment (biodiversity index, vegetation stratification richness, water feature proportion, microclimate comfort); (2) spatial organization (functional zoning clarity, pathway coherence, edge transition naturalness, node distribution rationality); (3) sensory experience (visual diversity, soundscape quality, tactile richness, olfactory intensity); and (4) social interaction (flexibility of social spaces, cultural inclusivity, intergenerational interaction potential, universal accessibility). Quantitative evaluation employed a 5-point Likert scale (1 = completely non-conforming; 5 = fully conforming), supplemented by field measurement data. Four representative cases (two international, two domestic) were systematically assessed through triangulated methods: on-site measurements (30 sampling points per case), user questionnaires (100 valid responses per case), expert ratings (five independent landscape architects), and behavioral observations (seven consecutive days). Qualitative analysis followed grounded theory methodology, incorporating semi-structured interviews (*n* = 40) and field observations to identify emergent patterns in user experience and design efficacy. The evaluation process comprised three phases: (a) preparatory stage (team establishment and protocol standardization), (b) field assessment (integrating spatial and temporal sampling), and (c) data analysis, including SPSS reliability testing (Cronbach’s α = 0.87), confirming strong internal consistency. Nonetheless, three methodological limitations were acknowledged: reliance on approximately 40% subjective evaluations, the need for cross-cultural validation, and limited longitudinal effect assessment.

The four cases selected for detailed study include two international examples—Jurong Lake Gardens in Singapore and the Cleveland Clinic Healing Garden in the United States—and two domestic Chinese cases—the "Mind Garden" in Shenzhen Bay Park and the Liangzhu Hospital Healing Garden in Hangzhou. These cases, representing diverse scales, functions, and cultural contexts, provide a comprehensive empirical foundation for this research and enable systematic comparative analysis of healing landscape practices across different settings.

### 4.2 Analysis of international case studies

#### 4.2.1 Singapore's Jurong Lake Gardens

Jurong Lake Gardens, Singapore’s first national garden integrating ecological restoration with mental health promotion, exemplifies post-pandemic healing landscape design for large public spaces. Completed in 2019 on a 90-hectare site, the project underwent functional adjustments during the pandemic, ultimately serving as a vital "green lung" for the city. Designed by Ramboll Studio Dreiseitl, the garden applies a "Nature Therapy" approach, employing classical tropical horticultural landscapes to create therapeutic environments that emphasize interactivity and immersive experiences.

Ecologically, the project not only conserved but also expanded the existing wetland ecosystem, supporting the proliferation of over 140 native plant species and more than 50 bird species through rainwater harvesting and natural filtration systems. The innovative "floating wetland" further enhanced biodiversity while enabling visitors to engage directly with aquatic ecosystems. Spatially, the design introduces a gradual transition from expansive public areas to secluded contemplative spaces, guiding visitors through layered natural experiences along winding wood-composite boardwalks. Key features include the "Bamboo Maze", offering semi-enclosed environments for meditation, and the open "Lawn Theatre", designed for communal gatherings, together creating a dynamic rhythm between active and passive spatial experiences.

In terms of sensory experience, the project incorporates a "Five Senses Garden" that engages multiple modalities through carefully selected plant species and spatial features. The herb garden includes fragrant plants such as lavender and jasmine to stimulate olfactory perception; the "Echo Valley" utilizes distinctive topographical acoustics to create auditory experiences; and the tactile exploration zone offers diverse textures for sensory engagement through touch. Social interaction is further fostered through the "Healing Community Program", which organizes outdoor yoga sessions, nature art workshops, and guided forest-bathing excursions, thereby integrating built and social dimensions to enhance the garden’s overall therapeutic benefits.

Post-occupancy surveys have revealed that visitor interaction within the garden saw a 36% increase during the pandemic, with 92% reporting a "considerable reduction in stress levels" (Rammo et al., 2022). The cause of the effect can be attributed to the garden’s comprehensive design strategy in fusion with ecological restoration, multisensory engagement, heterogeneous arrangements in space, and citizen participation to form a genuine urban therapeutic space (Wang et al., 2022; Chen et al., 2023).

#### 4.2.2 Cleveland Clinic Healing Garden

The Healing Garden at the Cleveland Clinic, completed in 2021 and spanning 1.2 hectares, represents a significant example of healing landscape design within healthcare settings. Developed by OLIN Studio, the project directly applies Roger Ulrich’s Supportive Environment Theory to create restorative spaces for patients, families, and medical staff. In collaboration with healthcare professionals, the design team introduced a "Symptom-Based Landscape Intervention" model, tailoring environmental experiences to address specific medical conditions, including post-operative recovery, chronic pain management, and mental stress reduction.

The spatial organization follows a "Nested Refuge" model, establishing hierarchies of space that vary in privacy through vegetative screening, terrain modulation, and pavilion structures. This arrangement provides users with a spectrum of choices ranging from fully open to highly secluded environments, enabling adjustment of social interaction in accordance with psychological needs. Particular emphasis is placed on accommodating patients with ambulatory impairments, with universal accessibility ensured through a barrier-free layout and climate-controlled garden platforms. Moreover, all major experiential areas are visible from patient room windows, allowing even bedridden patients to benefit from the therapeutic qualities of the surrounding landscape.

The planting strategy integrates therapeutic functions with seasonal experience, forming a "Therapeutic Plant Palette" in which calming species such as lavender and energizing species such as sunflowers are purposefully distributed across treatment zones. Sensory stimulation is a central design element, exemplified by the "Sound Garden", where wind-activated plants such as bamboo and bluebells, complemented by water features, generate ambient sounds distinct from medical equipment noise. Similarly, the "Tactile Labyrinth" offers varied textures through diverse plant species and materials, facilitating sensory integration therapy.

The initiative underscores the importance of flexibility in the post-pandemic context by promoting interpersonal communication within the constraints of social distancing through adaptable seating arrangements and distributed spatial configurations. Empirical evidence further demonstrates the therapeutic efficacy of such environments: surgical patients recovering in garden settings exhibit an 18% higher recovery rate compared to those in traditional indoor environments, require 12% less analgesic medication, and contribute to a 24% reduction in staff burnout, reflecting the benefits of supportive healing landscapes (Abd Elrahman, 2021; Bettaieb and Alsabban, 2021).

### 4.3 Analysis of Chinese domestic case studies

#### 4.3.1 Shenzhen Bay Park "Mind Garden"

The "Mind Garden", situated within Shenzhen Bay Park and opened in 2020 during the pandemic, is China’s first urban public space specifically designed to optimize mental well-being, covering an area of 5.6 hectares. Developed by the Turenscape Design Group, the project integrates traditional Chinese garden principles with contemporary psychological insights to create a culturally resonant therapeutic environment. Central to the design is an "Emotional Guidance" system that leads visitors on a psychological journey—from stress relief, to calmness, and ultimately to invigoration—through a sequence of seven linear nodes strategically embedded within the landscape.

The spatial configuration of the landscape draws upon traditional Chinese garden principles of "layered progression" and "meandering paths to seclusion", yet reframes these concepts through the lens of contemporary environmental psychology rather than traditional philosophy or aesthetics. The "Mountain and Water Imagery Area" employs classical borrowed-scenery techniques to integrate the vastness of Shenzhen Bay into the garden via carefully composed visual corridors, fostering both spatial expansiveness and psychological release. The "Bamboo Shadow and Breeze Area" creates semi-enclosed spaces through dense vegetation, generating shifting light-and-shadow effects that evoke introspection and solitude. By contrast, the "Water Mirror Gallery" utilizes the reflective and acoustic properties of water to orchestrate a multisensory and interactive experience.

The project emphasizes traditional Chinese sensory experiences by engaging all five senses. The "Pine Wave Listening Area" generates natural soundscapes through the interaction of pine trees and ocean winds, masking urban noise and fostering tranquility. The "Herb Tracking Area" features traditional Chinese medicinal plants such as chrysanthemum, ginkgo, and mint, combining olfactory stimulation with health education. Complementing these is the "Taste Garden", which incorporates edible plants and traditional foods that serve both nutritional and medicinal purposes, offering seasonal harvesting experiences that link cultural practice with therapeutic engagement.

Socially, the project introduced flexible "Co-cultivation Spaces" that accommodated diverse activities such as tai chi, qigong, and group psychological counseling. In response to the pandemic, these areas were adapted into outdoor classrooms and small-group activity zones that supported safe social distancing. Data monitoring revealed that, during this period, garden occupancy rates surpassed those of other public spaces by 41%, with an average stay time of 86 min—more than double the typical park average of 35 min. Serving as vital psychological "safe havens" for Shenzhen residents, the gardens significantly enhanced user well-being, with 93% of participants reporting improved mood following engagement in garden activities (Peters and Halleran, 2021; Elsaid and Ahmed, 2021).

#### 4.3.2 Hangzhou Liangzhu Hospital Healing Garden

The Liangzhu Hospital Healing Garden is a unique example of Chinese medical landscapes, blending traditional cultural symbolism with modern healing practices. The 0.6-hectare facility was completed in 2021. Led by Shu Landscape Design, the project is based on principles that integrate the "Five Elements Theory" inherent in Traditional Chinese Medicine with evidence-based design principles from modern Western studies, thus creating a healing setting with cultural relevance to Chinese patients. In the design process, the designers not only applied knowledge learned from Western research on medical landscapes but also worked closely with Traditional Chinese Medicine practitioners to reinterpret traditional health cultivation principles into a contemporary landscape language.

The architectural design was guided by a concept called "Five Qi Gardens", representing the five basic elements—metal, wood, water, fire, and earth—in five interconnected therapeutic environments. The active "Wood Area," defined by lush bamboo and pliable vegetation, supports light exercise and breathing control. The calming "Water Area" creates a contemplative mood through the use of moving water and reflective surfaces. The welcoming "Fire Area" provides natural light and communal spaces that promote social interaction. The stabilizing "Earth Area" features a medicinal garden that reaches into the realm of horticultural therapy. The refreshing "Metal Area" utilizes an open space approach that supports deep breathing and airflow circulation. This design allows patients with varying psychological disorders to choose environments that suit their personal requirements, thus supporting the principle of "dialectical therapy" expressed within Traditional Chinese Medicine.

The project lays strong emphasis on the fusion between traditional and modern-day technologies, and the "Digital Chinese Medicine Garden", where over 40 commonly used Chinese medicinal herbs are grown, provides a prime example. The space shares botany knowledge and medicinal attributes through digital boards to become an interactive educational space on the topic of health. At the same time, space serves as the basis for the hospital-based horticultural therapy program where patients participate in the growth of medicinal herbs to improve feelings of engagement and efficacy in themselves.

The spatial design considers the varied needs of different patient groups, with the "Tranquility Courtyard" designed especially for oncology patients, with increased privacy and comfortable shade to allow for emotional expression. In contrast, the "Vitality Circle", located near the rehabilitation department, includes paths and balancing equipment with increasingly difficult levels to prolong the benefits of physical therapies. Empirical studies have shown that engagement in garden activities led to an average 8% decrease in patients' mean arterial blood pressure, with 16% of patients reporting less self-assessed pain, 23% reporting less anxiety, and a 31% improvement in satisfaction among healthcare staff, highlighting the success of this therapeutic landscape that combines Chinese and Western approaches. This project is an innovative prototype for healing landscapes in the context of Chinese domestic medicine, blending traditional cultural wisdom with modern medical treatment.

### 4.4 Case comparison and evaluation

Through a systematic comparative analysis of international and Chinese healing landscape cases, this study identifies both key differences and shared characteristics, offering valuable insights for post-pandemic design practice. International cases are generally grounded in explicit evidence-based frameworks—for instance, the Cleveland Clinic Healing Garden directly applies Roger Ulrich’s Supportive Garden Theory and related clinical research—whereas Chinese cases more often integrate traditional cultural concepts with modern scientific knowledge. A notable example is the Liangzhu Hospital Healing Garden, which combines the Traditional Chinese Medicine Five Elements Theory with contemporary environmental psychology, illustrating a distinctive form of cultural adaptability.

In applying ecological design elements, international cases generally exhibit higher biodiversity indices and more complex habitat structures, with available data indicating an average plant species count approximately 1.7 times greater than that of Chinese cases (Menatti and Casado da Rocha, 2016). However, these quantitative claims warrant further substantiation through transparent methodological details and validation. By contrast, Chinese cases display more advanced microclimate regulation strategies, offering distinct advantages in enhancing comfort under monsoon climatic conditions. A comparison of spatial organization reveals that international cases typically employ clearly defined functional zoning and linear experiential sequences, whereas Chinese cases favor more fluid, overlapping spatial arrangements, reflecting the traditional garden principle of changing views with each step.

The comparative analysis of sensory experience design indicates that international cases typically place more emphasis on the precise design of tactile and olfactory experiences, such as the Cleveland Clinic's "Tactile Labyrinth"; Chinese cases invest more attention in visual sequences and soundscape design, as exemplified by the carefully designed visual screening and progressive revelation strategies in Shenzhen Bay's "Mind Garden." This reflects differences in sensory preferences across cultural backgrounds, providing important references for cross-cultural healing landscape design (Alhusban et al., 2022; Azuma et al., 2020; Quaglio et al., 2021; Alhadedy and Gabr, 2022).

In the comparison of social interaction elements, international cases tend to design more small-scale, distributed social spaces, emphasizing intimate conversations and small group interactions; while Chinese cases retain a larger proportion of collective activity venues, reflecting cultural needs for community collective activities. This difference demonstrated varying advantages during pandemic response: the decentralized design of international cases provided more flexibility during strict social distancing restrictions, while Chinese cases better supported community rebuilding activities after pandemic control relaxation.

The comprehensive evaluation indicates that successful healing landscapes, regardless of their cultural background, exhibit four common characteristics: systemic ecological thinking, progressive spatial experiences, diverse sensory stimulation, and inclusive social support. Post-pandemic healing landscape design should creatively adapt according to local cultural needs and climate conditions while drawing on international advanced experiences, achieving true "global thinking, local action."

## 5 Design strategies

### 5.1 Design principles and guiding philosophy

The establishment of therapeutic landscapes in response to the pandemic requires an interdisciplinary framework that synthesizes up-to-date research findings with tested environmental design principles. A thorough examination of case work and recent literature identifies five core principles forming the foundation to successful healing landscape design and translates to a range of scales and contexts.

The theory of "Biophilic Integration" argues that the intrinsic significance of the relationship between humans and nature is the fundamental mechanism for achieving healing. This theory extends beyond simple greening, embracing environmental features, natural forms and shapes, inherent patterns and processes, as well as light, space, and location-based relationships, together with evolved human-nature interactions. For post-pandemic applications, biophilic design has a strong focus on immune system support through interaction with beneficial environmental microbiomes and phytoncides from plant life.

The concept of "Adaptive Resilience" understands that functional healing spaces need to have the ability to adapt to changing situations such as climatic conditions, public health needs, or changing user requirements. This adaptive approach eschews rigid solutioning and instead advocates using modular and adaptable systems that can be rearranged in response to changes in conditions with regenerative design measures that maximize both ecologically driven and socially driven adaptiveness.

The "Sensory Calibration" theory assumes that healing occurs through definite sensory interfaces and requires careful attention to the intensity, variety, and sequencing of environmental stimulation. Sensory Calibration theory elaborates on the Attention Restoration Theory and expands its scope to encompass an extensive set of sensory experiences and acknowledges that different healing effects will be related to different sensory patterns—i.e., using stimulating settings to treat depressive states and using soothing settings to treat anxiety.

The social granularity principle underscores the need to have a variety of social settings that allow varying levels of interaction spanning from group settings to complete solitude. The principle recognizes the deep social and psychological impacts caused by the pandemic and gives support to a range of social activities that ensure psychological restoration through adequate levels of social engagement.

The concept of "Cultural Resonance" is inherently important because it acknowledges that healing processes are shaped by interpersonal relationships and cultural contexts. As such, healing environments ought to encompass culturally resonant elements that reflect users' identities, promote their sense of belonging, and support restorative connections to places through cultural and temporal continuity.

### 5.2 Urban-scale healing landscape design strategies

The growth of therapeutic spaces in urban environments requires concerted efforts to redesign urban infrastructures to turn spaces into integrated networks that support recovery. From assessments through case analysis and current research activities, four core strategies have been found to greatly improve mental well-being and overall well-being in urban places.

The "Network Therapeutic" model redresses urban green spaces as a networked system of healing landscapes to form a therapeutic network of landscapes that spread recuperative activities in the urban space. The strategy includes locating nodal points to enhance healing landscapes substantially, in addition to the establishment of green corridors that serve in multiple capacities as both ecological corridors and therapeutic pathways. The successful implementation would require mutual collaboration among agencies to integrate healing landscapes into transport systems, waterway management systems, and regulation policies. The successful implementation in Singapore's Park Connector Network provides evidence of the substantial benefits from well-planned green corridors that maximize opportunities to have restorative encounters; empirical data register higher wellbeing scores among residents living within 300 meters away from such connectors at 23% points than those who do not have such availability.

The "Microhealing" approach prioritizes the redesign of small, often overlooked urban spaces into individual therapeutic landscapes that support "restorative moments" in and among everyday urban activities. Unlike traditional pocket parks, microhealing sites are carefully designed to yield direct psychological respite through targeted sensory engagement. Implementation typically involves identification of parcels less than 0.2 hectares near high-stress urban environments, followed by the integration of intensive biophilic design elements, compelling auditory environments, and intentional manipulation of the microclimate.

The "Therapeutic Waterscapes" framework leverages urban water systems as integral therapeutic resources, informed by empirical research demonstrating that blue spaces yield considerable restorative benefits. This strategy prioritizes public access to waterfront areas, daylighting of buried urban streams, and incorporation of aquatic elements into stressful urban areas. Successful implementation of this strategy requires collaboration between landscape architects, water experts, and public health professionals to maximize water features for therapeutic use through thoughtful sensory design and improved accessibility.

The "Resilient Healing Infrastructure" model incorporates healing landscape functions into critical urban infrastructure, thus creating environments with multiple benefits, improving climate resilience while, at the same time, promoting mental wellness. The approach reimagines conventional infrastructure projects—like stormwater control, flood protection, and urban heat island abatement systems—into healing landscapes that provide psychological benefits in addition to their specified technical function.

### 5.3 Community-scale healing landscape design strategies

At the community level, healing landscape strategies focus on creating environments that are open and responsive to the diverse needs of neighborhoods and that foster social cohesion. The following four strategies have been particularly effective in creating healing environments that respond to community needs during the post-pandemic era.

The vision of the "Neighborhood Healing Hub" seeks to establish multifunctional neighborhood centers with therapeutic landscapes and situated in easily walkable proximity to residential areas. The hubs would be immersive spaces that evoke the senses, comprising multiple sensory zones that address varied restoration needs. Implementing this would necessarily involve participatory design processes to ascertain the exact healing needs and concern areas in the community and then developing adaptive spaces that can evolve in response to changing community needs. Prominent examples, including the Superblock program in Barcelona, illustrate that transforming 3-5% land in the neighborhood into dense healing landscapes can greatly improve restoration experiences; tests have found a 17% decrease in stress biomarkers among the neighborhood population.

The "Therapeutic Wayfinding" process utilizes intrinsic navigation systems within a community to promote mobility within restoration-oriented landscapes. It marries traditional urban legibility components—pathways, nodes, districts, edges, and landmarks—with contemporary understanding about organizing restoration environments. The process begins with the mapping of existing community stressors and existing restoration opportunities and then proceeds with designing interconnected pathways that consider carefully chosen sensory attributes to steer individuals toward restoration-prone environments. The process is particularly beneficial to populations with fractured green infrastructure because it maximally leverages the therapeutic potential of existing resources through carefully crafted links.

The "Inclusive Healing" strategy ensures that healing environments at the community level are specially designed to address the diverse needs and capacities of diverse users. The approach uses universal design to address the unique requirements of dis-advantaged groups including those with mobility impairment, sensory processing differences, cognitive impairment, and mental sensitivities. Its successful implementation requires systematic interaction with diverse user populations and the application of supportive environments theory in matching the psychological capacities with the intricacies of the environments.

The "Community Care Landscapes" program combines therapeutic horticulture with nature-based social programming in public open spaces. The model redevelops underutilized landscapes as active healing environments by enabling programs that promote environmental stewardship and public well-being. In order to realize this model effectively, it is necessary to create flexible landscape architecture to accommodate therapeutic programs—community gardens, forest bathing, and outdoor mindfulness, among others—and to educate community facilitators to deliver health-promoting programming.

### 5.4 Site-scale healing landscape design strategies

At the site level, healing landscapes require careful analysis in relation to spatial organization, material selection, and experiential qualities that have direct effects on human physiology and mood. The following methods outlined seek to illustrate research-based healing environments at the site level, allowing for the creation of viable healing environments.

The "Restorative Microzonation" system arrays site configurations into configurations to promote distinctive healing spaces to serve to enable targeted therapeutic outputs. The system extends the conventional microclimate design concept through the incorporation of four primary dimensions to a restorative experience that includes separation, scope, intrigue, and alignment. Implementation requires the configuration of a graduated range of spaces with diverse levels of enclosure, sensory stimulation, and social interaction opportunities to allow users to choose spaces in line with their psychological requirements. Empirical evidence indicates that spaces with at least three distinct microzones have much better performance in stimulating therapy than spaces with a homogeneous characteristic since users experience a 26% improvement in relief from stress benefits through their ability to find spaces attuned to their temporary psychological states.

The "Sensory Orchestration" approach utilizes empirically supported sensory design principles to design multi-sense healing spaces that activate the senses of vision, hearing, touch, smell, and proprioception. The approach goes beyond conventional aesthetic methods in that it deliberately designs sensory elements to cause pre-defined physiological and psychological responses. The strategy requires the rigorous sensory mapping and integrated design strategy taking into account visual complexity, acoustic conditions, tactile interaction, olfactory experiences, and kinasesthesia. Successful projects such as the Nacadia Healing Forest in Denmark offer evidence that well-designed multi-sense spaces can cause a decrease in cortisol levels up to 23% after 30 min exposure.

The "Temporal Design" strategy unifies dynamic changes in multiple dimensions—like daily cycles, seasonal changes, and long-term ecological developments—to maximize the potential for restoration. The approach acknowledges the requirement for therapeutic landscapes to engage users actively through changing conditions rather than providing set environments. The implementation includes the integration of elements with distinctive seasonal characteristics, design characteristics providing varied lighting conditions during the day, and the institution of vegetation maintenance patterns that produce desired changes over a duration of time. Empirical research proves that settings with discernible, yet unobtrusive changes considerably enhance fascination and attention restoration compared to immutable settings.

The "Material Biophilia" approach employs current research into human psychological and physiological responses to natural materials and patterns. This approach extends the foundations of biophilic design to the level of specific material selection and arrangement, incorporating biophilic design patterns such as the material's connection to nature, complexity and order, the prospect and refuge concepts, and mystery elements. The implementation of this approach involves highlighting natural materials with visible grain structures, incorporating fractal elements at multiple scales, and creating a dynamic tension between ordered and organic forms.

## 6 Conclusion

This study investigates the development of therapeutic landscapes in urban, community, and site-specific contexts during the pandemic, identifying key design elements and strategies that enhance healing environments. The findings reveal that therapeutic landscapes incorporate four primary components: (1) ecological considerations, (2) spatial organization, (3) sensory engagement, and (4) social interaction. These elements collectively foster holistic environments aimed at promoting human well-being.

A comparative analysis of international and Chinese case studies reveals that therapeutic landscapes exhibit universal characteristics—such as ecologically integrated systems, carefully planned spatial sequences, multisensory facilities, and adaptable spaces promoting social interaction—despite cultural and contextual differences. However, the study identifies significant variations in implementation, particularly in how cultural context influences sensory appeal, spatial composition, and social programming.

The design guidelines derived from this study offer a systematic framework to assist landscape architects, urban planners, and health professionals in implementing therapeutic landscapes across various scales—from urban systems to site-specific interventions. Emphasizing flexibility, accessibility, and cultural relevance, these guidelines address the complex health needs of contemporary society. A phased implementation approach, applied at multiple scales, facilitates integrated strategies for health promotion through landscape design.

While the research in hand presents a significant breakthrough in understanding healing garden design in post-pandemic environments, several limitations need to be acknowledged. The long-term performance of such spaces requires longitudinal research, and in particular, the impact on chronic conditions should be studied further. Design interventions' extensibility to different cultural and climatic conditions should be studied in depth too.

Future studies should examine the quantitative relationships between the various elements in design and the respective quantifiable effects on health, the integration of computer-based technologies into physical healing environments, and the development of standardized assessment protocols to determine the therapeutic effects of healing landscapes. Amidst continued societal responses to the effects of global health crises, healing landscapes become critical resources for promoting community resilience and overall well-being through carefully designed environments that support reconnection among people, the natural environment, and oneself.

## Data Availability

The original contributions presented in the study are included in the article/supplementary material, further inquiries can be directed to the corresponding author.
